# The Rice Abscisic Acid-Responsive RING Finger E3 Ligase OsRF1 Targets OsPP2C09 for Degradation and Confers Drought and Salinity Tolerance in Rice

**DOI:** 10.3389/fpls.2021.797940

**Published:** 2022-01-13

**Authors:** Suyeon Kim, Seong-Im Park, Hyeokjin Kwon, Mi Hyeon Cho, Beom-Gi Kim, Joo Hee Chung, Myung Hee Nam, Ji Sun Song, Kyung-Hwan Kim, In Sun Yoon

**Affiliations:** ^1^Gene Engineering Division, National Institute of Agricultural Sciences, Rural Development Administration (RDA), Jeonju, South Korea; ^2^Metabolic Engineering Division, National Academy of Agricultural Science (NAAS), Rural Development Administration (RDA), Jeonju, South Korea; ^3^Seoul Center, Korea Basic Science (KBSI), Seoul, South Korea

**Keywords:** RING finger, PP2CA protein degradation, ABA signaling, stress tolerance, rice (*Oryza sativa*)

## Abstract

Drought and salinity are major important factors that restrain growth and productivity of rice. In plants, many really interesting new gene (RING) finger proteins have been reported to enhance drought and salt tolerance. However, their mode of action and interacting substrates are largely unknown. Here, we identified a new small RING-H2 type E3 ligase *OsRF1*, which is involved in the ABA and stress responses of rice. *OsRF1* transcripts were highly induced by ABA, salt, or drought treatment. Upregulation of *OsRF1* in transgenic rice conferred drought and salt tolerance and increased endogenous ABA levels. Consistent with this, faster transcriptional activation of key ABA biosynthetic genes, *ZEP, NCED3*, and *ABA4*, was observed in *OsRF1*-OE plants compared with wild type in response to drought stress. Yeast two-hybrid assay, BiFC, and co-immunoprecipitation analysis identified clade A PP2C proteins as direct interacting partners with OsRF1. *In vitro* ubiquitination assay indicated that OsRF1 exhibited E3 ligase activity, and that it targeted OsPP2C09 protein for ubiquitination and degradation. Cell-free degradation assay further showed that the OsPP2C09 protein is more rapidly degraded by ABA in the *OsRF1*-OE rice than in the wild type. The combined results suggested that OsRF1 is a positive player of stress responses by modulating protein stability of clade A PP2C proteins, negative regulators of ABA signaling.

## Introduction

Drought is one of the most influential environmental problems in plant productivity. Frequent occurrences of drought and abnormal weather events have been observed lately all over the world. Tons of crops are damaged by drought, which is caused by abnormal weather events. Along with the predictable increasing population in the world and decreasing bias of available water and field for crop production, it is necessary to make an effort to establish strategy for improvement of crop yield under water-limiting conditions. In rice, there have been noticeable attempts to cope with drought stress through overexpression of drought-resistant genes (Fang and Xiong, 2015; Li et al., 2017; Shen et al., 2017; Yuan et al., 2019; Wang et al., 2020; Xu et al., 2020). Most of them are related to increasing of abscisic acid (ABA) responses.

The plant hormone ABA regulates major processes in response against various biotic and abiotic stresses (Lee and Luan, 2012; Nakashima and Yamaguchi-Shinozaki, 2013; Yoshida et al., 2014). ABA signaling pathways are regulated by three protein families, pyrabactin resistance 1 (PYR1)/PYR1-like (PYL)/regulatory components of ABA receptors (RCAR), clade A protein phosphatase type 2Cs (PP2Cs), and sucrose non-fermenting 1-related kinase 2 (SnRK2) (Ma et al., 2009; Park et al., 2009; Santiago et al., 2009; Nishimura et al., 2010). Under ABA-limited conditions, clade A PP2C proteins bind to SnRK2 and inhibit its phosphorylation activity. These binding and inactivation of SnRK2 block ABA signaling and result in inhibition of ABA-dependent responses. When plants face environmental stresses such as drought, the endogenous ABA level increases, and ABA binds to PYR1/PYL/RCAR receptors. ABA-bound PYR1/PYL/RCAR receptors form a complex with PP2Cs, preventing the inhibition of SnRK2. SnRK2 is activated by auto-phosphorylation and activates downstream elements such as bZIP transcription factors and S-type anion channels, subsequently leading to strong ABA signal transduction and responses such as stomatal closure (Ng et al., 2011; Soon et al., 2012; Osakabe et al., 2014).

Really interesting new gene (RING) finger proteins are a subgroup of zinc-finger protein superfamily that contains a RING domain possessing a novel consensus motif of cysteines and histidines (Freemont et al., 1991; Hanson et al., 1991). Previous genomic analyses have revealed that RING finger proteins in plants are large members of plant proteins. In Arabidopsis and rice, 469 and 425 proteins, respectively, were predicted as containing one or more RING domains (Kosarev et al., 2002; Stone et al., 2005; Lim et al., 2010). Most RING finger proteins in plants could be subcategorized into RING-H2 (C3H2C3) or RING-HC (C3HC4), depending on existence of Cys or His on their fifth conserved residue in RING motif (Lim et al., 2010). Also, some other minor RING subfamilies, such as RING-v, RING-D, RING-S/T, RING-G, and RING-C2, have been reported in Arabidopsis, but only the RING-v and RING-C2 families were found in rice (Lim et al., 2010).

Most RING-finger proteins are predicted to act as an E3 ubiquitin ligase, which is a major component of protein ubiquitination process (Deshaies and Joazeiro, 2009). Ubiquitination is proceeded by enzymatic process of three enzymes, ubiquitin activating enzyme (E1), ubiquitin conjugating enzyme (E2), and ubiquitin ligase (E3) (Deshaies and Joazeiro, 2009; Metzger et al., 2014). The E3 ubiquitin ligase decides the specificity of ubiquitination by binding to ubiquitinated E2 and a substrate and transferring ubiquitin from E2 to the substrate. Transferred monoubiquitin could be elongated by the E2-E3 complex, causing proteasome-dependent proteolysis (Deshaies and Joazeiro, 2009).

In plants, many RING E3 ligases have been reported to be involved in abiotic stress responses and ABA signaling (Ko et al., 2006; Lee et al., 2011; Zeng et al., 2014; Wu et al., 2016; Brugiere et al., 2017). For example, *Arabidopsis XERICO* (*AtXerico*) and its homologs of rice (rice RING-H2 finger protein 1, *OsRHP1*), corn (*ZmXerico1*), and poplar (*Ptxerico*) conferred drought tolerance by increasing the biosynthesis of endogenous ABA when they were overexpressed (Ko et al., 2006; Zeng et al., 2013, 2014; Kim M.H. et al., 2020). Along with *AtXerico*, *AtAIRP3*/*AtAIRP4* (ABA-insensitive RING protein), *SDIR1* (salt and drought-induced RING finger1 protein), and *RHA2a*/*RHA2b* (Arabidopsis RING-H2) is positively involved in the regulation of ABA signaling (Zhang et al., 2007; Li et al., 2011; Yang et al., 2016). In rice, a number of RING finger proteins have been recently reported to play either negative or positive roles in the regulation of drought and salinity stress (Gao et al., 2011; Ning et al., 2011; Hwang et al., 2016; Park et al., 2018; Park et al., 2019b; Kim M.H. et al., 2020; Qin et al., 2020; Kim et al., 2021b; Kim and Jang, 2021; Seo et al., 2021). So far, only a few protein targets of RING finger E3 ligases have been identified. For example, ZmXerico1 targets the protein stability of ABA hydroxylases, leading to enhance ABA levels and drought stress resistance (Brugiere et al., 2017). In *Arabidopsis*, RING finger E3 ligases, RING DOMAIN LIGASE (RGLG)1, RGLG5, PP2CA-interacting RING finger protein (PIR1), and AtAIRP4 were reported to be regulating ABA signaling by degradation of PP2CA proteins (Wu et al., 2016; Baek et al., 2019). *Arabidopsis* SDIR1 protein targets the PCD/DOH (DIMERIZATION COFACTOR OF HEPATOCYTE NUCLEAR FACTOR1) homolog protein SDIRIP1 and EIN3-binding F-box protein (EBF1/EBF2) to regulate ABA and ethylene signaling under environmental stress conditions (Zhang et al., 2015; Hao et al., 2021). In rice, several protein targets for RING finger E3 ligases were recently reported. The *Oryza sativa* cytoplasmic-localized RING finger protein 1 (OsCLR1), *Oryza sativa* salt-induced RING finger protein 4 (OsSIRP4), and *Oryza sativa* As-induced RING E3 ligase3 (OsAIR3) targeted stem-specific OsTSJT1 proteins, OsPEX11-1 and OsMOT1 3, for ubiquitination and degradation, respectively (Park et al., 2019a; Kim et al., 2021a; Kim and Jang, 2021). However, target proteins and molecular mechanism for most rice RING finger proteins remain to be unveiled.

Here, we report a new RING-H2 E3 ligase, *OsRF1*, which plays as a positive regulator of drought and salt stress responses in rice. The overexpression of *OsRF1* resulted in an ABA-hypersensitive phenotype and induced the salt and drought tolerance of rice. The stress tolerance of *OsRF1*-OE transgenic rice correlated with ABA accumulation and ABA hypersensitivity. We further demonstrated that OsRF1 targets a clade A protein phosphatase, OsPP2C09, a core negative regulatory component of ABA signaling components, for ubiquitination and protein degradation. This result suggests that *OsPP2C09* is one of the molecular linkages of *OsRF1* function in ABA-dependent stress tolerance. Finally, we showed that *OsRF1* attenuates GA-induced degradation of *Slender1* (SLR1) protein, a key repressor of GA signaling pathway. These finding provide further understanding of a new rice RING finger member participating in ABA-GA signaling network for growth regulation in rice under environmental stress conditions.

## Materials and Methods

### Plant Materials, Transformation, and Growth Conditions

The rice cultivar *Oryza sativa* cv Dongjin was used in this study. To generate *OsRF1* overexpressing rice (*OsRF1*-OE), we used the codon-optimized synthetic ORF of *OsRF1* (LOC_Os09g30160), because it was difficult to clone *OsRF1* by RT-PCR because of high GC content. The nucleotide sequence of synthetic *OsRF1* had 75.5% identity with the wild-type *OsRF1* sequence, and the translated amino acid sequence was 100% identical to that of OsRF1 (Supplementary Figure 1). The synthetic *OsRF1* was cloned into the pCAMBIA1300 vector for constitutive expression of the gene under the control of CaMV 35S promoter. Rice callus derived from the seeds of *O. sativa* Dongjin was transformed with *Agrobacterium tumefaciens* LBA4404 harboring the overexpression vector, as previously described (Hiei et al., 1994). Transgenic calli were selected, and shoots were regenerated in 1/2 Murashige and Skoog (MS) media in the presence of 30 μg/ml hygromycin. Insertion and expression of the transgene in *OsRF1*-OE lines were validated by reverse transcription PCR (RT-PCR) analysis with primers specific to the synthetic *OsRF1* sequence. T_2_ or T_3_ plants of three independent transgenic lines (*OsRF1*-OE1, *OsRF1*-OE2, *OsRF1*-OE3) were used for further analyses. Rice plants were grown in soil in a green house or precisely controlled growth room for drought and salt tolerance assay.

To analyze gene expression, growth responses to hormone treatment, and ABA contents, rice seedlings were grown in 1/2 MS agar medium [per liter: 4.4 g MS salt, 30 g sucrose, 0.5 g 2-(N-morpholino) ethanesulfonic acid (MES), 8 g plant agar, pH 5.8] in a growth room at 28°C with long day condition (16 h: 8 h, light:dark cycle). For hormone treatment, rice seeds were germinated in distilled water for 3 days, transferred into 1/2 MS media containing 1 μM gibberellin (GA_3_) or 3 μM abscisic acids (ABA), and grown at 28°C under long day conditions. After 7–10 days, total shoot length and second leaf sheath length of each seedling were measured.

### Immunoblot Analysis of *Slender1* Protein

For detection of endogenous rice DELLA protein level, an antibody against rice SLR1 protein was purchased from Cosmo Bio (Cat. No. CT-NU-001-1; Tokyo, Japan). *OsRF1*-OE, *OsPP2C09*-OE, and wild type seedlings were grown in 1/2 MS media containing GA_3_or ABA. The *OsPP2C09*-OE transgenic rice lines were kindly provided in Min et al. (2021). Whole shoots were ground in liquid N_2_, and total protein was extracted using an extraction buffer containing 0.05 M Tris–HCl (pH 7.4), 0.2% SDS, 5% glycerol, 1.5% TritonX-100, 1% β-mercaptoethanol, 1 mM EDTA, 1 mM dithiothreitol, and 1X cOmplete™ Mini EDTA-free Protease Inhibitor (Roche, Rotkreuz, Switzerland). A total of 30 μg of proteins were dissolved in 10% SDS-PAGE gel and used for immunoblot analysis. The protein-segregated SDS-PAGE gels were transferred to a PVDF membrane and incubated with anti-SLR1 or anti-actin (Abcam, Cambridge, MA, United States) antibodies and then with peroxidase conjugated anti-rabbit (Thermo Fisher Scientific, Waltham, MA, United States) according to the manufacturer’s protocol. Peroxidase activity was detected using SuperSignal West Femto Maximum Sensitivity Substrate (Pierce, Rockford, IL, United States).

### Measurement of Abscisic Acids Content

Ten-day-old rice seedlings treated with 200 mM NaCl for 24 h or air-dried for 2 and 4 h were ground using a mortar and pestle after exposure to liquid nitrogen, and 200 mg of the powder was transferred to a microcentrifuge tube. The samples were extracted using 1 ml of an extraction solvent (25 mM KH2PO4, pH 3.0/methanol, 20/80) and 20 ng of [2H6]-2-*cis*-4-trans ABA (OlChemin Ltd., Olomouc, Czechia) as an internal standard, and then centrifuged at 13,000 rpm for 20 min at 4°C. The supernatant was dried and dissolved in 100 μl of 80% MeOH. ABA analysis was performed with a Finnigan TSQ LC/MS/MS system consisting of a Finnigan Surveyor LC pump, Finnigan Surveyor refrigerated autosampler, and Finnigan TSQ Quantum Ultra EMRtriple quadrupole tandem mass spectrometer (Thermo Fisher Scientific, San Jose, CA, United States). Chromatographic separation was performed using an extend-C18 column (2.1 mm × 150 mm, 5 μm; Agilent Technologies, Santa Clara, CA, United States). The mobile phase consisted of solvent A (deionized water: 0.1% acetic acid) and solvent B (acetonitrile). The gradient was applied at a flow rate of 0.2 ml/min as follows: solvent B was equilibrated from 0 to 2 min with 5% B, and then it was linearly increased to 100% for 10 min. Finally, its composition was reduced to 5% for 15 min and equilibrated at 5% B for 5 min. We injected 20 μl of the sample into the HPLC column. All mass analyses were performed by electrospray ionization (ESI) in negative mode. Analysis of ABA was based on selected reaction monitoring (SRM) of ion pairs using mass transition 263.00–153.13 for ABA and 269.00–159.14 for [2H6]-ABA.

### Quantitative Real-Time PCR Analysis

For *OsRF1* expression analysis, WT rice seedlings were grown for 10 days in 1/2 MS agar media and treated with 10 μM ABA for 6 h, 200 mM NaCl for 6 h, and drought stress for 2 and 4 h. Before salt or ABA treatment, the rice seedlings were pre-incubated in 1/2 MS liquid media at 28°C overnight under 100% relative humidity conditions. To analyze the expression of ABA synthesis gene under salt and drought conditions, the 10-day-old WT and *OsRF1*-OE rice seedlings were challenged with 200 mM NaCl for 6 h or dried for 1, 2, or 4 h. Total RNA was extracted from the shoot of rice seedlings using RNeasy Plant Mini Kit (QIAGEN, Valencia, CA, United States). The extracted RNA samples were then treated with Amplification Grade DNase I (Invitrogen, Carlsbad, CA, United States) to eliminate genomic DNA. The first-strand complementary DNA (cDNA) was synthesized from 2 μg of total RNA with Superscript Reverse Transcriptase III (Invitrogen, Carlsbad, CA, United States). cDNA 0.5 μl was used as a template in 20 μl of reaction containing 0.5 nM of desired primers and AccuPower 2 × Greenstar qPCR Master Mix (Bioneer, Daejeon, South Korea). The transcripts were quantified using 7,500 Real Time PCR System (Applied Biosystems, Foster City, CA, United States) according to the manufacturer’s protocol. The *OsUbi5* gene (LOC_Os01g22490) was used as an internal standard for normalization of cDNA concentration variations. The primer sequences used in this study are listed in Supplementary Table 1.

### Drought and Salt Tolerance Analysis

The seeds of WT and *OsRF1*-OE lines were planted in 1/2 MS media or 1/2 MS containing 30 μg/ml hygromycin for 7 days, respectively, and seedlings were transferred and grown in Hyponex media (Hyponex Japan, Osaka, Japan) for 3 days. Ten seedlings of WT and each *OsRF1*-OE line were transplanted in the same soil pot and grown for 2 weeks in the green house. To evaluate the salt tolerance of *OsRF1*-OE, 22-day-old WT and *OsRF1*-OE rice seedlings were supplied with a 200-mM NaCl solution for 12 days and then re-watered for 7 days for recovery. For drought tolerance assay, the rice seedlings were grown under water-limited conditions for 7 days followed by recovery for 7 days. To quantify the extent of damage under salt and drought stress, the damage state of rice seedlings was scored from 0 (non-damaged) to 4 (fully damaged) according to the appearance of visual symptoms such as yellowing, rolling, and drying of leaves. The damage score and image of rice plants were taken before treatment, after treatment, and after recovery.

### Water Loss Assay

To compare the leaf water loss rate of WT and *OsRF1*-OE rice, 40-day-old plants were incubated overnight under 100% relative humidity conditions in order to maintain maximum water content. After incubation, two or three young leaves in the same developmental stage were taken from each plant and air-dried. Changes in leaf weight were measured for 7 h with an interval of 1 h. The experiments were performed with three independent biological repeats.

Water use dynamics of the WT and *OsRF1*-OE rice at the whole plant level was assessed using DroughtSpotter (Phenospex, Heerlen, Netherlands) as described by Park et al. (2021). Briefly, a single plant was grown in a pot for 3–4 weeks and loaded on a DroughtSpotter cell located in a precisely controlled environmental room operated under a 14-h light/10-h dark photoperiod with 50% humidity between 30°C (at noon) and 23°C (at midnight). Before the start of the experiment, the weight of each pot was equalized to 620 g with soil and water, and each pot was irrigated between 20:00 and 20:30 of the day. Drought stress was imposed by stopping water supply for 3 days, and the weight of each pot was automatically measured every 1 min. An empty soil pot without plant was used as a reference to estimate the effect of the ambient condition on water evaporation from the soil. Reading data were combined over 90-min periods, and the plant water loss rate (mg/min/cm^2^) of individual plants was calculated according to the formula below. The average water loss rate of four individual plants of wild-type and *OsRF1*-OE lines was calculated in biological repeat experiments.

Whole plant water loss rate (mg/min/cm^2^) = [(plant pot weight loss every 90 min)–(empty pot weight loss every 90 min)]/90/plant area.

### Yeast Two-Hybrid Assay

For yeast Y2H assay, full-length CDSs of synthetic *OsRF1* was cloned into the pGBKT7 vector of Matchmaker Gal4 Two-Hybrid System 3 (Clontech, Palo Alto, CA, United States). pGADT7 vectors fused with nine OsPP2CAs (OsPP2C06, OsPP2C08, OsPP2C09, OsPP2C30, OsPP2C49, OsPP2C50, OsPP2C51, OsPP2C53, and OsPP2C68) were kindly provided by Dr. B-G. Kim (National Institute of Agricultural Sciences, South Korea). Bait and prey constructs were co-transformed into the *Saccharomyces cerevisiae* AH109 strain (*MATa*, *trp1-901*, *leu2-3*, *112*, *ura3-52*, *his3200*, *gal4*Δ, *gal80*Δ, *LYS2*:*GAL1UAS-GAL1TATA-HIS3*, *GAL2UAS-GAL2TATA-ADE2*, *URA3:MEL1UAS-MEL1TATA-lacZ*, *MEL1*) using the lithium acetate method, and the transformants were selected in SD minimal media (Clontech, Palo Alto, CA, United States) lacking Leu and Trp (SD-LT). For identification of protein interaction, the transformants were spotted in SD-LT media, such as 5-bromo-4-chloro-3-indolyl-a-D-galactopyranoside (X-α-Gal) and SD media lacking Leu, Trp, and His (SD-LTH), and grown at 30°C for 3 days.

### Subcellular Localization and Biomolecular Fluorescence Complementation Analysis in Rice Protoplasts

The synthetic codon-optimized CDSs of *OsRF1* and *OsRHP1* (LOC_Os08g38460) were cloned in the pENTR/D/TOPO vector (Invitrogen, Carlsbad, CA, United States) and cloned in-frame of the 5′ end of the GFP (smGFP) in the pGEM-GFP vector. Transient expression in rice protoplast was determined by PEG-mediated transformation, as previously described (Bhatnagar et al., 2017). For further analysis of the localization of OsRF1 protein, the ER mCherry marker CD3-960 was co-expressed in the rice protoplast with OsRF1-GFP or OsRHP1-GFP proteins. GFP and mCherry fluorescence were observed under a confocal laser scanning microscope (Leica TCS SP8; Leica, Wetzlar, Germany).

To carry out BiFC assays, synthetic CDSs of *OsRF1* were transferred from pENTR/D/TOPO to pVYCE vector, resulting in fusion with the C-terminus of yellow fluorescent protein (YFP). The N-terminus of YFP-fused OsPP2C53 (LOC_Os05g51510), OsPP2C50 (LOC_Os05g46040), OsPP2C09 (LOC_Os01g62760), and OsPP2C30 (LOC_Os03g16170) pVYNE vectors were kindly provided by Dr. B-G. Kim (National Institute of Agricultural Sciences, South Korea). The constructs were co-transformed into protoplast and incubated for 16 h with 50 μM Z-Leu-Leu-Leu-aldehyde (MG132; Sigma-Aldrich, St. Louis, MO, United States) to prevent 26S proteasome-mediated protein degradation. Florescence signals within the protoplasts were observed under the confocal laser scanning microscope, as described above.

### Co-immunoprecipitation Assay

For Co-immunoprecipitation assays, *OsPP2C09* was cloned into the pGEM-UbiHA vector, and the synthetic CDS of *OsRF1* was cloned into the pGEM-GFP vector. Rice protoplasts were co-transfected with these constructs and incubated for 20 h in the presence of 10 μM MG132. After incubation, the samples were centrifuged at 100 × *g* for 10 min at RT, and the supernatant was removed. The cells were suspended in a Co-IP buffer [150 mM NaCl, 50 mM Tris–HCl (pH 7.5), 0.5% NP40, 0.5% Triton X-100, 1 mM EDTA, 2 mM EGTA, 2 mM MgCl_2_, 10 μM MG132, and 1X protease inhibitor cocktail] and sonicated. The samples were centrifuged at 12,000 rpm at 4°C, and the supernatant was mixed with GFP-Trap beads (Chromotek, Planegg, Germany) and incubated at 4°C for 2 h. The beads were washed with a washing buffer [150 mM NaCl, 50 mM Tris–HCl (pH 7.5), 1 mM EDTA, 2 mM EGTA, 2 mM MgCl_2_, 10 μM MG132, and 1X protease inhibitor cocktail], and then an SDS sample buffer was added. The immunoprecipitation products were detected by SDS-PAGE and western blot using anti-HA (Sigma-Aldrich, St. Louis, MO, United States) or anti-GFP antibody (Life Technologies, Carlsbad, CA, United States).

### Recombinant Protein Purification

The synthetic CDS of *OsRF1* was cloned into the pGEX-4T vector using the Gateway cloning system, resulting in a glutathione S-transferase (GST)-tagged *OsRF1*-overexpressing plasmid. The construct was transformed into *Escherichia coli* BL21 (DE3) pLysS (Invitrogen, Carlsbad, CA, United States). For OsPP2C09 protein purification, the ORF of *OsPP2C09* gene (LOC_Os01g62760) was cloned into the pET28 (a) vector and transformed into *E. coli* Rosetta (DE3) cells (Invitrogen, Carlsbad, CA, United States). The *E. coli* cells were grown at 37°C to OD600 of 0.5 and induced using 100 μM isopropyl β-D-1-thiogalactopyranoside (IPTG) at 18°C for 20 h. A pellet of 100 ml culture was resuspended with 10 ml of a protein incubation buffer [100 mM Tris-Cl (pH 8.0), 500 mM NaCl, and 5% glycerol], supplemented with 1X cOmplete™, Mini, EDTA-free Protease Inhibitor (Roche, Rotkreuz, Switzerland), and sonicated at 40% of amplitude on ice with five cycles of 15 s sonication and 45 s rest. The sonicated cells were centrifuged at 12,000 rpm for 20 min at 4°C, and the supernatant was collected and diluted with a protein incubation buffer up to 50 ml. The lysate from *OsRF1* or *OsPP2C09* overexpressing cells was loaded in 1 ml of Glutathione Agarose 4B (Peptron, Daejeon, South Korea) or Ni-NTA Agarose (QIAGEN, Valencia, CA, United States), and GST-tagged OsRF1 and His-tagged OsPP2C09 were purified by affinity chromatography method according to the manufacturer’s protocol. The protein concentration was determined by Bradford method and the purified protein fractions were stored at −80°C until use.

### *In vitro* Ubiquitination Assay

For self-ubiquitination assay, each 30 μl of a reaction mixture contained 500 ng of purified recombinant OsRF1-GST, 50 ng of human E1-activating enzyme (Sigma-Aldrich, St. Louis, MO, United States), 200 ng of recombinant human ubiquitin-conjugating enzyme (E2) UbcH5b (Enzo Life Sciences, Farmingdale, NY, United States), and 10 μg of bovine ubiquitin (Sigma-Aldrich, St. Louis, MO, United States) in a reaction buffer [50 mM Tris-Cl (pH 7.4), 5 mM MgCl_2_, 3 mM ATP, and 0.5 mM DL-dithiothreitol (DTT)] and incubated at 30°C for 2 h. For *in vitro* E3 ubiquitination assay, 500 ng of recombinant OsPP2C-His protein was incubated with GST-OsRF1 in the reaction mixture as described above. The OsPP2C50-His recombinant protein was kindly provided by Dr. B-G. Kim (National Institute of Agricultural Sciences, South Korea). The reaction was stopped by mixing with a 5X SDS loading buffer [250 mM Tris-Cl (pH 7.4), 10% SDS (w/v), 0.5% bromophenol blue (w/v), and 50% glycerol] and 100 mM DTT. The samples were dissolved in 10% SDS-PAGE gels and immunoblotted using anti-GST (Santa Cruz Biotechnology, Santa Cruz, CA, United States), anti-His (Invitrogen, Carlsbad, CA, United States), or anti-ubiquitin (Sigma-Aldrich, St. Louis, MO, United States) antibodies.

### Cell-Free Degradation Assay

To investigate the protein stability of OsPP2C09 in the *OsRF1*-OE1 and WT rice plants, seedlings grown in 1/2 MS media for 1 week in a growth room in a 14-h light/10-h dark cycle were treated with 100 μM ABA for 24 h under the same growth conditions. Total proteins were extracted in a degradation buffer containing 25 mM Tris–HCl (pH 7.4), 10 mM NaCl, 10 mM MgCl_2_, 5 mM DTT, 3 mM ATP, and EDTA-free protease inhibitor, and adjusted to equal concentrations with the degradation buffer. Protein extracts (30 μg) were incubated with His-tagged OsPP2C protein (500 ng) in the presence or absence of 50 μM MG132 for 0, 1, 1.5, and 2 h at 28°C. The protein samples were loaded on 12% SDS-PAGE gels, blotted into a PVDF membrane (Atto Corp., Tokyo, Japan), and immunoblotted with anti-His-tagged OsPP2C09 antibody and anti-Actin antibody (Agrisera, Vannas, Sweden). For detection of immunoreactive proteins, SuperSignal West Femto Maximum Sensitivity Substrate (Thermo Fisher Scientific, Waltham, MA, United States) and Fusion SL Gel Detection System (Vilber Lourmat, Marne-la-Vallée, France) were used.

## Results

### Identification of a New RING H2-Type Protein, *OsRF1*, in Rice

**FIGURE 1:**
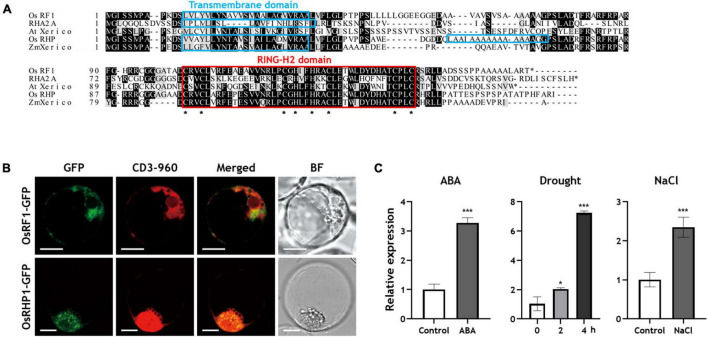
Sequence alignment analysis, subcellular localization, and expression profiles of OsRF1. **(A)** Deduced amino acid sequence alignment of OsRF1 and other RING-H2 proteins. The predicted amino acid sequence of OsRF1 was aligned with Arabidopsis RING-H2 group A (RHA) 2A (NM_1013778), AtXerico (NP_178507), rice OsRHP1 (NP_001062121), and ZmXerico (NP_001151741) using Clustal Omega (https://www.ebi.ac.uk/Tools/msa/clustalo/). The red box and asterisks indicated RING-H2 domains and conserved Cys and His residues, respectively. Putative transmembrane domains were predicted using TMHMM2.0 (http://www.cbs.dtu.dk/services/TMHMM-2.0/) and indicated as light blue box. Identical (black boxes) and similar (gray boxes) sequences were also indicated. **(B)** Subcellular localization of OsRF1 and OsRHP1. The GFP-tagged OsRF1 and OsRHP1 were co-expressed with an ER mCherry marker, CD3-960, and visualized by confocal microscopy. The GFP and mCherry signals are represented in green and red, respectively. Bar = 10 μm. **(C)** Expression of *OsRF1* under various conditions. Ten-day-old seedlings were treated with 10 μM ABA for 6 h, 200 mM NaCl for 6 h, and drought stress for 2 and 4 h. The relative expression level of *OsRF1* in shoots was analyzed by qRT-PCR with the ΔΔCt method. The expression level of each gene was normalized with the expression level of the internal control gene, *Ubiquitin 5*. Data represent mean ± standard deviation (SD) from three independent experiments with three biological replicates (*n* = 3). Statistical analysis of data was performed by Student’s *t*-test. *P < 0.05, ***P < 0.005.

Previous studies have reported that the overexpression of RING finger proteins, such as *AtXerico*, confers drought tolerance to plants (Ko et al., 2006; Zeng et al., 2013, 2014). In rice, a total of 425 genes were predicted to encode a RING finger protein by *in silico* analysis, and OsRHP1, a RING-H2 type of protein, was reported as an *AtXerico* homologue that gave rice drought and salt tolerance when it overexpressed (Lim et al., 2010; Zeng et al., 2014). In the present study, we identified a novel rice RING finger protein OsRF1 which has high similarity in amino acid sequence with AtXerico. The predicted OsRF1 protein was composed of 165 amino acids and has 17.06 kD and 8.23 of calculated molecular weight and pI, respectively^1^. The OsRF1 protein shows a relatively high amino acid sequence identity (68%) with OsRHP1 and low identity (34%) with AtXerico, but contains the conserved canonical RING-H2 domain, suggesting that OsRF1 is a small RING-H2 protein that could act as an E3 ubiquitin ligase (Figure 1A).

Transmembrane domain analysis using TMHMM 2.0^2^ revealed that OsRF1 has a putative transmembrane domain in the N-terminal region (13–35 residues), which is similar to that of *Arabidopsis* RING-H2 group A (RHA) 2A and *Zea mays* ZmXerico (Figure 1A). In OsRHP1, two transmembrane domains (13–35 and 55–74 residues) were predicted, and the first one (13–35 residues) was also similar to other RING–H2 proteins. These data suggested that these two RING-H2 proteins are membrane-anchored proteins. Regarding the function of the transmembrane domain, one previous report has shown that the N-terminal 40 aa transmembrane domain of ZmXerico protein was sufficient to target ER localization (Brugiere et al., 2017). To determine the subcellular localization of *OsRF1* and *OsRHP1*, we constructed GFP-fused OsRF1 and OsRHP1 proteins, and observed their GFP signals by confocal microscopy (Figure 1B). Fluorescent signals were observed in the cytoplasm, peripheral region of the nucleus, and showed a stipple appearance in the protoplast membrane. We further showed that the GFP-fused proteins were co-expressed with an mCherry endoplasmic reticulum (ER) marker (CD3-960) (Nelson et al., 2007). Taken together, we concluded that OsRF1 is likely to be anchored to the ER membrane.

### Expression of *OsRF1* Was Regulated by Abscisic Acid-Dependent Manners

**FIGURE 2:**
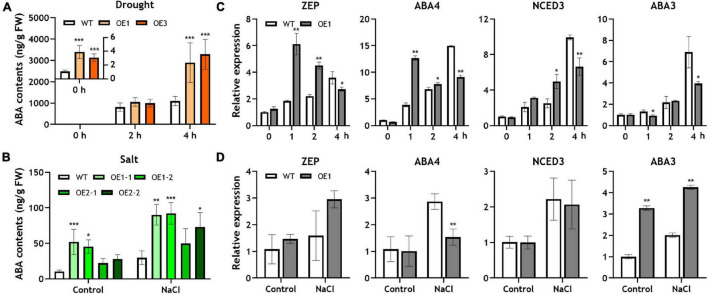
Endogenous ABA concentration and expression of ABA biosynthetic genes in the *OsRF1*-OE plant under drought and salt stress. **(A,B)** Endogenous ABA contents in WT and *OsRF1*-OE rice under drought and salt stress conditions. The ABA content in shoots was measured by TSQ LC/MS/MS. Data represent mean (± SD) from three independent experiments with two biological replicates (*n* = 3). **(C,D)** qRT-PCR analysis of ABA biosynthetic genes in the *OsRF1*-OE plant under salt and drought stress. The relative expression levels of ABA-related genes in shoots of stressed or non-stressed wild-type and *OsRF1*-OE were indicated as bar graph. The expression levels of each gene were normalized with expression levels of the internal control gene, *Ubiquitin,5* and analyzed with the ΔΔCt method. *ZEP*, zeaxanthin epoxidase; *NCED*, 9-*cis*-epoxycarotenoid dioxygenase; *ABA3*, molybdenum cofactor (MoCo) sulfurase. Data represent mean (± SD) from three independent experiments with three biological replicates (*n* = 3). All statistical analyses for data were performed by Student’s *t*-test. *P < 0.05; **P < 0.01; ***P < 0.005.

A large number of plant RING-finger proteins have been reported to be participating in biological processes, especially stress responses (Lee et al., 2001, 2011; Hong et al., 2007; Zhang et al., 2007). The plant hormone ABA is deeply related to stress responses, and its level is increased under diverse stress conditions (Lee and Luan, 2012; Nakashima and Yamaguchi-Shinozaki, 2013). To understand the specific characteristics of *OsRF1*, we identified the expression properties of *OsRF1* under ABA and stress conditions. When 10-day old rice seedlings were treated with ABA for 6 h, the expression level of *OsRF1* increased by 3.25-fold (Figure 1C). When the rice seedlings were treated with drought stress, *OsRF1* transcript level increased by up to sevenfold, depending on stress-challenging time (Figure 1C). Similarly, its expression level increased by 2.25-fold after 6 h of exposure to 200 mM NaCl condition (Figure 1C). ABA contents analysis revealed that endogenous ABA levels of rice rapidly increased when treated with drought stress or NaCl (Figures 2A,B). This suggests that the expression of *OsRF1* is regulated by ABA under stress conditions, and that it could have a role in drought and salt stress response. We further examined the spatiotemporal gene expression pattern of *OsRF1* using the RiceXPro database. The data indicate that the expression of *OsRF1* is not strictly restricted to specific tissues, although higher expression was observed in reproductive organs than in vegetative tissues (Supplementary Figure 2).

### *OsRF1*-OE Rice Represented Abscisic Acid-Hypersensitive Phenotype

To characterize the function of *OsRF1*, we constructed *OsRF1*-overexpressed transformed rice (*OsRF1*-OE). The synthetic CDS of *OsRF1* was fused with a CaMV 35S promoter in CAMBIA1300 vectors and transferred into *O. sativa* L. cv. Dong-jin *via Agrobacterium*-mediated transformation, as described in Materials and methods. The insertion of *OsRF1* was validated by RT-PCR analysis, indicating a high expression level of the synthetic *OsRF1* gene (Supplementary Figure 3A). Homozygous T_2_ or T_3_*OsRF1*-OE plants were used for further studies.

**FIGURE 3:**
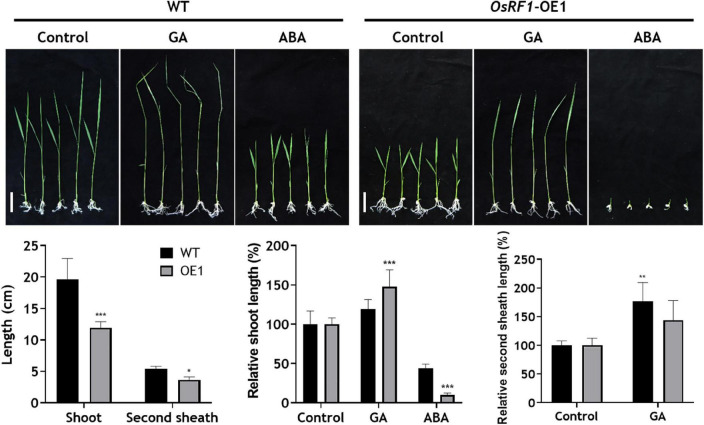
Overexpression of *OsRF1* leads to enhanced sensitivity to ABA-mediated inhibition of shoot elongation. WT and *OsRF1*-OE rice were germinated in distilled water for 3 days and transplanted to 1/2 MS media containing 1 μM GA_3_ or 3 μM ABA. The total shoot length and second internode length of rice seedlings were measured after 7–10 days. Bar = 2 cm. Data represent the mean (± SD) from three biological replicates (*n* = 25 for each line). All statistical analyses for data were performed by Student’s *t*-test. *P < 0.05; **P < 0.01; ***P < 0.005.

The overexpression of *OsRF1* results in growth retardation and a semi-dwarf phenotype in the paddy field (Supplementary Figure 3B). Additionally, the seed size of the *OsRF1*-OE rice decreased, whereas seed dormancy was enhanced (Supplementary Figures 3C–E). It has been previously reported that rice dwarfism and seed dormancy are closely correlated with the hormonal disturbance of GA or ABA (Ueguchi-Tanaka et al., 2000; Itoh et al., 2004; Zhu et al., 2005). To dissect the effect of *OsRF1* overexpression on plant growth, we measured the total shoot and second sheath length of rice seedlings under hormone treatment conditions (Figure 3). When the WT and *OsRF1*-OE rice were grown for 7 days in 1/2 MS media without hormones, the total shoot length of *OsRF1*-OE was about 58.2% greater than that of WT (Figure 3). Under GA treatment conditions, the second sheath length increased in both WT and *OsRF1*-OE, but the growth ratio in the second sheath between the GA_3_-treated group and the control group in the *OsRF1*-OE seedlings was significantly lower (*P* < 0.05) than that in the WT seedlings (Figure 3). This implicates that the *OsRF1*-OE seedlings is less sensitive to GA compared to the WT rice. In contrast, the growth of *OsRF1*-OE seedlings was completely inhibited in the presence of 3 μM ABA. The ratio of total shoot length between the ABA-treated and control groups was much lower in *OsRF1*-OE compared to WT, indicating that *OsRF1*-OE was hypersensitive to ABA treatment (*P* < 0.005).

### Overexpression of *OsRF1* Enhanced Drought and Salt Stress Tolerance *via* Increasing Abscisic Acid Levels in Rice

**FIGURE 4:**
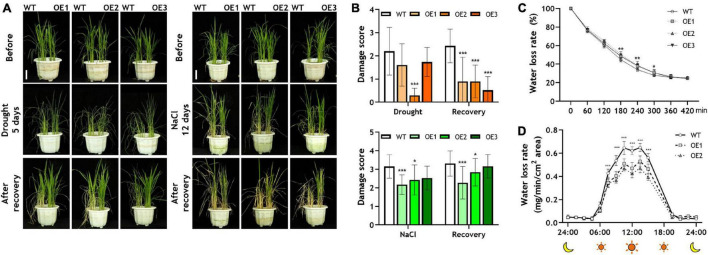
Drought and salt responses of wild-type and *OsRF1*-OE lines. **(A)** Drought and salt tolerance assay of transgenic rice. The WT and *OsRF1*-OE lines, which were grown for 10 days in 1/2 MS medium, were transplanted in soil pots and grown for 12 days. For salt tolerance assay, 22-day-old WT and *OsRF1*-OE rice seedlings were grown in soil pots with 200 mM NaCl for 12 days and then without NaCl for 7 days. For drought tolerance assay, 22-day-old seedlings were grown without water for 5 days and then with water for 7 days. Bar = 5 cm. **(B)** Damage rates of leaves after stress and recovery was measured and scored from 0 to 4. Data represent the mean (± SD) from three independent experiments with two biological replicates (*n* = 10 for each line). **(C)** Water loss assay in detached leaves of wild-type and *OsRF1*-OE lines. Water loss rate of detached leaves of 40-day-old WT and *OsRF1*-OE transgenic rice was measured. Data represent mean (± SD) from three biological repeats with seven or eight replicates of each line. **(D)** Whole plant water loss rate of wild-type and *OsRF1*-OE lines. Water loss rate was automatically measured from a soil pot planted with a single rice plant using DroughtSpotter under the none irrigating (NONE) mode. Data represent mean (±SD) from two independent experiments with five plants of each line. Two-way ANOVA and Fisher’s LSD test were performed by comparison with WT plants as controls. *P < 0.05; **P < 0.01; ***P < 0.001.

To identify whether *OsRF1* has a role in drought and salt stress responses, we carried out a drought and salt tolerance assay of three independent *OsRF1*-OE plants (Figure 4A). The 22-day-old *OsRF1*-OE rice seedlings were challenged with drought or 200 mM NaCl stress for 5 and 12 days, and recovered for 7 days (Figure 4A). To discriminate the damage level under stress, the damage rate of each plant was scored from 0 to 4 depending on the healthy state of leaves. Under drought stress, the damaging rate of *OsRF1*-OE was similar to that of WT, but *OsRF1*-OE recovered much faster than WT after re-hydration (Figure 4B, upper panel). In the case of salt stress, the rate of damaged leaves in *OsRF1*-OE was much lower than that in WT (Figure 4B, lower panel). For further identification of the effect of *OsRF1*-overexpression on drought stress-resistance, we measured the water loss rate of healthy leaves from 40-day-old *OsRF1*-OE lines under drought conditions (Figure 4C). Leaf water loss rate was determined by measuring the weight of detached leaves every 1 h until the weight did not change. The result from leaf water loss assay revealed that the water loss rate of leaves of *OsRF1*-OE lines was significantly lower than that of WT after 3 to 5 h (*P* < 0.05). We further investigated the water loss dynamics of *OsRF1*-OE lines at whole plant level using DroughtSpotter, an automated gravimetric platform for phenotyping the drought tolerance of a plant, as described by Park et al. (2021). In this DroughtSpotter experiment, fully watered individual plants were subjected to drought stress by stopping irrigation for 1–3 days, and the weight of each pot was automatically measured every 1 min. Water loss rates from individual plants were calculated as described in Materials and methods. As shown in Figure 4D, the water loss rate of the *OsRF1*-OE plants is significantly lower than that of the WT plants (*P* < 0.001).

The combined results from stress tolerance and water loss assays suggested that the overexpression of *OsRF1* could improve the tolerance to drought and salt stress by enhancing the water-keeping ability of leaves. Because *OsRF1* was ABA-inducible and ABA is deeply related to stress responses in plants, we hypothesized that the strong tolerance against drought and salt stress is due to different levels of ABA in *OsRF1*-OE that result in the semi-dwarf and ABA-hypersensitive phenotype of *OsRF1*-OE (Lee and Luan, 2012; Nakashima and Yamaguchi-Shinozaki, 2013). To clarify this hypothesis, we analyzed endogenous ABA contents in rice shoots, which were treated with drought or salt stress (Figures 2A,B). For drought stress treatment, 10-day-old seedlings grown in MS-agar media were pulled out and air-dried. Under non-stressed conditions, the ABA contents were very low, but ABA sharply increased by more than a thousand times when challenged with severe drought stress (Figure 2A). The ABA levels of *OsRF1*-OE were significantly higher than those of WT under the normal and drought stress conditions. The ABA contents of *OsRF1*-OE were 3.1-fold higher in average (*P* < 0.05) in the resting stage, and 2.82-fold higher on average (*P* < 0.05) under drought stress than those of the WT plants (Figure 2A). To impose salt stress, rice seedlings were treated with 200 mM NaCl for 24 h. Under salt stress, the ABA levels increased (2.92-fold on average) in the *OsRF1*-OE seedlings compared to WT (*P* < 0.05; Figure 2B). These results suggest that the overexpression of *OsRF1* increased the endogenous ABA level under normal and stressed conditions and induced drought and salt tolerance in rice.

In Arabidopsis and rice, it was reported that the overexpression of RING-H2 proteins, such as *AtXerico* and *OsRHP1*, induced the expression of ABA biosynthetic genes, which caused increase in endogenous ABA levels (Ko et al., 2006; Zeng et al., 2014). To identify whether the increased ABA levels in *OsRF1*-OE plants were due to induced ABA biosynthetic pathway or not, we investigated the expression of four genes related to ABA biosynthesis in rice treated with drought or salt stress. As shown in Figure 2C, the expression of genes encoding zeaxanthin epoxidase (*ZEP*), neoxanthin synthase (*NSY*; *ABA4*), and 9-*cis*-epoxycarotenoid dioxygenase (*NCED3*) is significantly increased under 1- and 2-h drought stress (Figure 2C). Under salt stress conditions, however, it is noteworthy that the expression of *ABA3*, which encodes molybdenum cofactor (MoCo) sulfurase, was significantly higher in *OsRF1*-OE than in WT, and that the expression of *ZEP*, *ABA4*, and *NCED3* was not different from that of WT (Figure 2D). In *Arabidopsis*, the ABA3 gene plays important roles in the biosynthesis of ABA, allantoin, and anti-oxidant metabolites for osmotic stress responses (Xiong et al., 2001; Watanabe et al., 2018). The reason for the discriminated expression profiles of ABA biosynthetic pathways in *OsRF1*-OE in our short-term salt and drought stress experiments remains to be addressed. Taken together, our data suggest that increased ABA levels in shoots of *OsRF1*-OE lines were correlated with the induced expression levels of ABA biosynthetic genes, and that ABA biosynthesis might be differently regulated according to the type of stress.

### OsRF1 Interacts With Clade A PP2C Proteins

**FIGURE 5:**
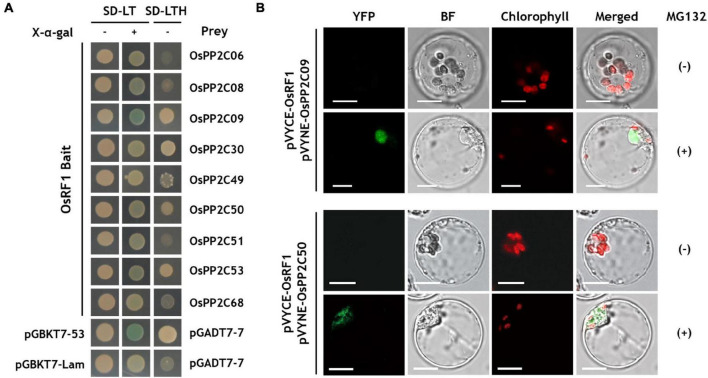
OsRF1 interacts with clade A OsPP2Cs. **(A)** Yeast two hybrid (Y2H) assay. AH109 yeast cells were co-transformed with the bait (pGBKT7-OsRF1) and prey (pGADT7-OsPP2Cs) constructs. Transformed cells were plated in color change selection media (SD-LT/X-α-Gal) or growth selection media (SD-LTH) and grown at 30°C for 3 days. **(B)** BiFC assay using pVYCE-OsRF1 and pVYNE-OsPP2Cs in rice protoplasts. The rice protoplast co-expressing OsRF1-VYCE and OsPP2C09-VYNE or OsPP2C50-VYNE in the presence or absence of MG132 was visualized using a confocal laser scanning microscope. YFP signals are represented in green, and chloroplast auto-fluorescence is represented in red. YFP; yellow fluorescent protein; BF, bright field. Bar = 10 μm.

Abscisic acid (ABA) signaling is regulated by three major components, PYR1/PYL/RCAR receptors, clade A PP2C (PP2CA) proteins, and SnRK2 (Ma et al., 2009; Park et al., 2009; Santiago et al., 2009; Nishimura et al., 2010). Based on the results of analyses of ABA contents and expression of ABA biosynthesis genes, we considered the possibility that *OsRF1* regulates the ABA signaling components. Therefore, we conducted Y2H assays to identify the interaction between OsRF1 and nine members of clade A OsPP2C proteins (Figure 5A). In SD-LTH media, yeast cells expressing OsRF1 fused to the GAL4 DNA-binding domain (BD) with OsPP2C09, OsPP2C30, and OsPP2C50, and OsPP2C53 fused to the GAL4 activation domain (AD) showed a strong interaction (Figure 5A). The Y2H analysis on SD-LT media containing X-α-gal indicated that there was a strong interaction of OsRF1 and OsPP2C09 and weak interaction between OsRF1 and OsPP2C08, OsPP2C30, OsPP2C50, and OsPP2C53. From the results of two Y2H analyses, OsPP2C09, OsPP2C30, OsPP2C50, and OsPP2C53 were selected as candidates interacting with OsRF1 and used for further analysis.

The protein-protein interactions of OsRF1 and OsPP2CA were confirmed by BiFC analysis in rice protoplasts (Figure 5B). pVYCE-OsRF1 strongly interacted with pVYNE-OsPP2C09 in the nucleus of the protoplast when incubated with MG132, a 26S proteasome inhibitor (Figure 5B, upper panel). Similar to pVYNE-OsPP2C09, pVYNE-OsPP2C50 exhibited YFP signal only under MG132-treated conditions, but the signal was observed in the cytoplasmic region (Figure 5B, lower panel). Min et al. (2019) surveyed the subcellular localization of nine OsPP2CA proteins in rice protoplasts and found that the OsPP2CAs were clearly divided into two groups: one with nucleus-specific and the other with non-nucleus specific localization (Min et al., 2019). OsPP2C09 is nucleus-specific, whereas the localization of OsPP2C50 was not restrained to the nucleus and distributed to the cytoplasm. Taken together, our results suggest that OsRF1 may interact with either cytoplasmic or nuclear substrates. Several E3 ligases have been known to translocate in response to environmental signals. For example, Lim et al. reported that a rice Golgi-localized OsHCI1 protein relocated to the nucleus and mediated the ubiquitination of nuclear-localized substrates under heat stress conditions (Lim et al., 2013a). At present, how the ER-anchored OsRF1 protein associates with the nuclear OsPP2C09 protein remains to be elucidated. One hypothesis is that the OsRF1 protein could be released to the cytoplasm by signal-dependent specific cleavage of the N-terminus transmembrane domain. Indeed, prediction of cleavage sites of different protease families using PROSPER (PROtease Specificity Prediction server^3^) showed that the transmembrane domain of OsRF1 is highly enriched with protease cleavage sites (Supplementary Figure 4).

Although OsPP2C53 and OsPP2C30 showed a binding signal with OsRF1 in the Y2H analysis, no fluorescence signals were observed in the protoplast expressing pVYNE-OsPP2C53 and OsPP2C30 with pVYCE-OsRF1 regardless of MG132 treatment (Supplementary Figure 5). The combined results indicated that the OsRF1 protein interacted with two OsPP2CA proteins, OsPP2C09 and OsPP2C50, suggesting that *OsRF1* could be a new member of ABA signaling regulators.

### OsRF1 Targets OsPP2C09 for Ubiquitination and Degradation

**FIGURE 6:**
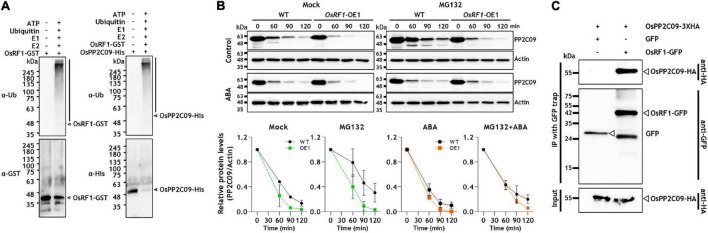
OsRF1 mediates ubiquitination and degradation of OsPP2C09. **(A)** *In vitro* self-ubiquitination of OsRF1 and *in vitro* ubiquitination of OsPP2C09 by OsRF1. Purified OsRF1-GST was incubated with the OsPP2C09-His protein in the presence or absence of human E1, human E2, and bovine ubiquitin (Ub), and incubated at 30°C for 2 h. The reaction samples were mixed with a 5X SDS-PAGE loading buffer, resolved in SDS-PAGE, and immunoblotted with anti-ubiquitin, anti-GST, and anti-His antibodies. The polyubiquitinated proteins are indicated as black bars. **(B)** Cell-free degradation assay of OsPP2C09. Protein extracts were prepared from seedlings of wild-type and *OsRF1*-OE lines treated with or without 100 μM ABA and incubated with purified OsPP2C09-His at 28°C supplemented at indicated time points. Anti-His antibody was used to detect the OsPP2C09-His protein. Representative data from three independent experiments. Quantification of anti-OsPP2C09-His and anti-Actin signal intensity using the Image J software. The protein levels of OsPP2C09-His in WT or *OsRF1*-OE extracts with indicated treatments at 0 min were set as 1. Data represent mean (± SD) from two independent experiments with three biological replicates (*n* = 3). **(C)** Co-immunoprecipitation assay of OsPP2C09. The OsRF1-GFP fusion protein was co-expressed with the OsPP2C09-HA fusion protein in rice protoplasts. Total protein extracts from the transformed protoplasts were pull-downed with GFP-trap beads and detected by immunoblot using anti-HA antibody and anti-GFP antibody.

Most RING fingers are expected to exhibit E3 ligase activity (Deshaies and Joazeiro, 2009). As described in Figure 1, the predicted OsRF1 protein contains a conserved RING-H2 domain, so we carried out a ubiquitination assay using a purified OsRF1-GST fusion protein to reveal whether the OsRF1 protein exhibits E3 ligase activity (Figure 6A). For *in vitro* auto-ubiquitination assay, the purified OsRF1-GST protein was incubated with human ubiquitin activating enzyme (E1), human ubiquitin conjugating enzyme (E2), and bovine ubiquitin (Ub) in a reaction solution at 30°C for 2 h. After incubation, polyubiquitination band was detected in the immunoblot using anti-ubiquitin and anti-GST antibodies, suggesting that OsRF1 could act as an E3 ligase (Figure 6A, left panel). In the absence of E1, E2, ATP, or ubiquitin, and when the purified GST protein was incubated in the reaction buffer without OsRF1, ubiquitination band was not detected, supporting that the appearance of polyUQ band was correlated with the E3 ligase activity of OsRF1. Next, we examined that OsPP2C09, a binding candidate for OsRF1, could be ubiquitinated by OsRF1. The ubiquitination assay with OsPP2C09 using anti-ubiquitin and anti-His antibodies showed ubiquitination activity when they were incubated with all ubiquitination components (Figure 6A, right panel). The clear polyubiquitinated band for OsPP2C09 suggested that it is a direct substrate of OsRF1. We also detected a polyubiquitinated band for OsPP2C50, suggesting that OsPP2C50 could serve as a substrate of OsRF1 for ubiquitination (Supplementary Figure 6A).

The E3 ligase is a component of protein ubiquitination that causes 26S proteasome-mediated protein degradation (Deshaies and Joazeiro, 2009; Metzger et al., 2014). The ubiquitination of OsPP2C09 by OsRF1 implied the idea that OsPP2C09 protein was degraded through OsRF1-mediated 26S proteasome degradation. To investigate the protein stability of OsPP2C09, we prepared protein extracts from *OsRF1*-OE and WT rice seedlings and, conducted a time-course cell-free degradation assay of His-tagged OsPP2C09 protein in the presence or absence of ABA. As shown in Figure 6B, ABA enhanced OsPP2C09 degradation, and OsPP2C09 protein levels were more unstable in the *OsRF*1-OE plants than in the WT plants. The degradation was delayed by MG132 treatment (Figure 6B), indicating that OsPP2C09 is degraded by the 26S proteasome. In addition, OsPP2C09 protein stability was decreased in the drought-stressed plants (Supplementary Figure 7). Our combined data suggest that OsRF1 is involved in OsPP2C09 protein degradation *in vitro*. On the other hand, we obtained unexpected results that the OsPP2C50 protein was much more stable than OsPP2C09 in the cell-free degradation assay (Supplementary Figure 6B). In addition, the degradation rate of OsPP2C50 was not significantly different between the *OsRF1*-OE1 and WT plants (Supplementary Figure 6B), suggesting that there may be additional factors controlling the degradation of the OsPP2C50 protein other than OsRF1. Therefore, OsPP2C09 and OsPP2C50 displayed a distinguishable pattern of spatiotemporal gene expression (Supplementary Figure 2), subcellular localization (Min et al., 2019), and protein stability (Supplementary Figure 6B). In this study, we further focused on the interaction of OsPP2C09 and OsRF1.

To verify the *in vivo* interaction of OsRF1 and OsPP2C09, we transiently co-expressed OsRF1-GFP and OsPP2C09-HA fusion proteins in rice protoplasts, and conducted a co-immunoprecipitation analysis. The results showed that OsRF1-GFP co-precipitated with the OsPP2C09-HA protein (Figure 6C). Together, our results indicate that OsRF1 directly interacts with OsPP2C09 for ubiquitination and degradation.

### Overexpression of OsRF1 and OsPP2C09 Affects the Stability of the SLR1 Protein

**FIGURE 7:**
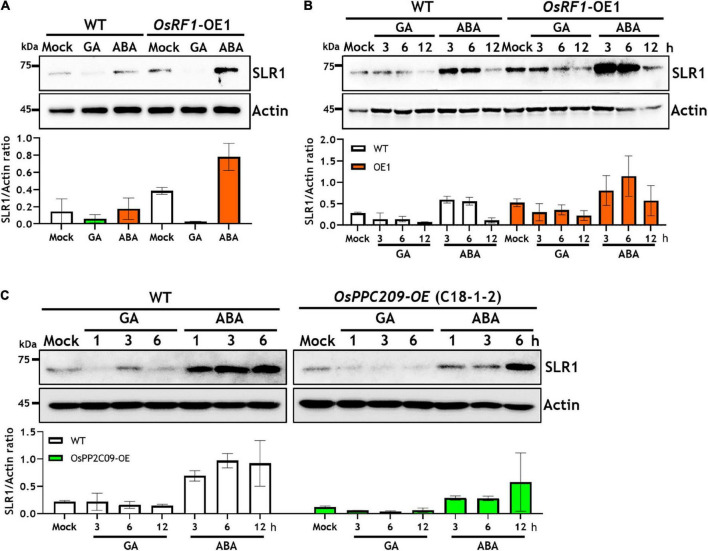
Overexpression of OsRF1 and OsPP2C09 affects the stability of Slender1 (SLR1) protein. **(A)** Protein level of endogenous SLR1 in WT and *OsRF1*-OE rice seedlings grown for 7 days in 1/2 MS agar media containing 50 μM GA_3_ or 50 μM ABA. **(B)** Protein stability of SLR1 in WT and *OsRF1*-OE rice in response to GA_3_ or ABA treatment. Seven-day-old seedlings exposed for 3 to 12 h in the presence of 50 μM GA_3_ or 50 μM ABA. **(C)** Protein stability of SLR1 in WT and *OsPP2C09*-OE rice in response to GA_3_ or ABA treatment. Seven-day-old seedlings were exposed for 1 to 6 h in the presence of 5 μM GA_3_ or 50 μM ABA. A total of 30 μg of whole protein extracts from rice shoots were dissolved in 10% SDS-PAGE and immunoblotted using anti-SLR1 antibody. Rice actin protein was used as loading control determined by immunoblotting using anti-Actin antibody. Quantification of anti-SLR1 and anti-actin signal intensity using the Image J software. SLR1 protein levels were normalized by anti-actin intensity. Data represent mean (±SD) from two independent experiments with three biological replicates (*n* = 3).

Along with enhanced stress tolerance and ABA hypersensitivity, the overexpression of OsRF1 in rice displayed growth retardation as well (Figure 3 and Supplementary Figure 3B). To obtain clues whether the growth retardation of *OsRF1*-OE is associated with ABA-GA interaction, we investigated the endogenous SLR1 protein levels in WT and *OsRF1*-OE lines under hormone-treated conditions (Figure 7A). SLR1 is a rice DELLA protein known as a key negative regulator that determines GA-responsive stem elongation and seed germination in rice. When the WT and *OsRF1*-OE seedlings were grown for 7 days in the hormone-containing 1/2 MS agar medium, a higher level of the SLR1 protein was detected in the *OsRF1*-OE seedlings than in WT under both unstressed and ABA-treated conditions (Figure 7A). Under GA_3_-treated conditions, the SLR1 protein was not detected in both the *OsRF1*-OE and WT plants, presumably because of complete depletion of the endogenous SLR1 protein under prolonged GA conditions (Figure 7A). We, therefore, investigated the time-dependent degradation of the SLR1 protein in response to 50 μM ABA or 50 μM GA_3_. Figure 7B shows that the SLR1 protein was rapidly degraded by GA_3_, and that the protein level was almost undetectable after 12 h of GA_3_ treatment in the WT seedlings. It appeared that the SLR1 protein was more stable in the presence of ABA (Figure 7B). Again, we found that the SLR1 protein level was much higher in the *OsRF1*-OE seedlings than in WT under GA_3_ or ABA-treated conditions (Figure 7B). The combined results suggest that *OsRF1* may restrain seedling growth by stabilizing the SLR1 protein through the ABA signaling pathway. As OsRF1 showed E3 ligase activity involved in ubiquitin-mediated protein degradation, it is plausible to assume that OsRF1 indirectly stabilized the SLR1 protein.

**FIGURE 8:**
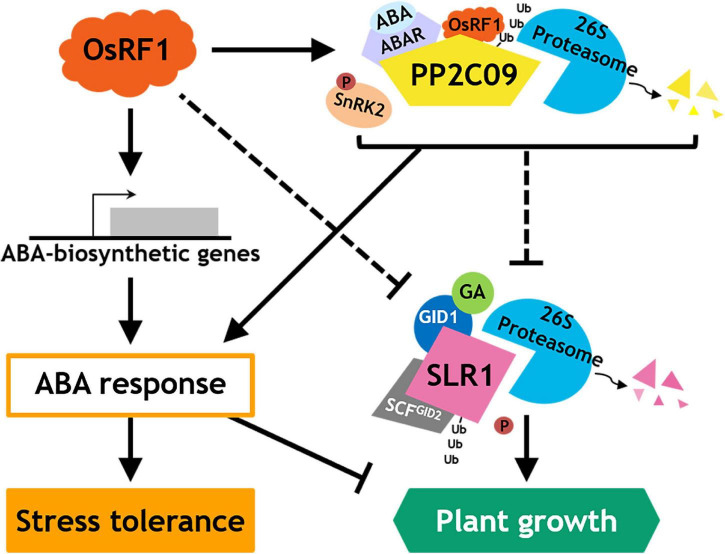
Proposed model for action of OsRF1 in ABA signaling, stress response, and growth regulation.

As we demonstrated that the OsRF1 targets a key repressor of ABA signaling OsPP2C09 (Figures 6A,B) and as previous reports indicated that the phosphorylation status was closely correlated with the protein stability of DELLA proteins (Wang et al., 2009; Dai and Xue, 2010; Qin et al., 2014; Blanco-Tourinan et al., 2020), we further investigated the SRL1 protein level in the OsPP2C09-OE transgenic rice. The *OsPP2C09*-OE transgenic rice was reported to display hypersensitive phenotypes in response to ABA, drought, and salinity (Miao et al., 2020; Min et al., 2021). Interestingly, Figure 7C clearly indicates that the SLR1 protein level was much lower in the *OsPP2C09*-OE transgenic rice than in the WT plants in the presence of 5 μM GA_3_ or 50 μM ABA. Therefore, the combined results implicate a close correlation between OsRF1 and OsPP2C09 in the antagonistic control of ABA signaling, SLR1 protein stability, growth response, and stress tolerance of rice (Figure 8).

## Discussion

The RING zinc finger proteins were characterized by the presence of the conserved RING domain which mediates protein-protein interaction and ubiquitination (Deshaies and Joazeiro, 2009). Previous studies on RING finger proteins have revealed that they are a large protein family in plants that participates in various biological process such as seed germination, regulation of cell cycles, and biotic and abiotic stress responses (Lee et al., 2001, 2011; Kosarev et al., 2002; Stone et al., 2005; Ko et al., 2006; Hong et al., 2007; Zhang et al., 2007; Liu et al., 2008; Bu et al., 2009; Lim et al., 2010; Park et al., 2010). In rice, a total of 425 genes were predicted to be encoding RING finger proteins from *in silico* analysis and expected to be related to abiotic stress responses (Lim et al., 2010, 2013b). A number of rice RING finger proteins have been reported to play either negative or positive roles in the regulation of drought and salinity stress. For example, the rice RING E3 ligases *Oryza sativa* salt-, ABA- and drought-induced RING finger protein 1 (*OsSADR1*), *Oryza sativa* drought-, heat-, and salt-induced RING finger protein 1 (*OsDHSRP1*), *Oryza sativa* drought-induced SINA protein 1 (*OsDIS1*), and *Oryza sativa* salt-induced RING finger proteins (*OsSIRP1* and *OsSIRP4*) reduced the tolerance for drought or salt stress when heterogeneously expressed in *Arabidopsis* (Hwang et al., 2016; Park et al., 2018; Kim J.H. et al., 2020; Kim and Jang, 2021). Among these, a negative regulatory role in drought response was further established for *OsDIS1*and*OsDIR1*in rice (Gao et al., 2011; Ning et al., 2011; Seo et al., 2021). In contrast to these diverse negative regulatory rice RING finger proteins, a few positive players were identified in the abiotic stress response of rice. The RING H2 type E3 ligase *OsSIRH2-14* and C4HC3 RING E3 ligase *OsRFPv6* enhanced salinity tolerance (Park et al., 2019b; Qin et al., 2020; Kim et al., 2021b). In addition, the molecular function and targets of most rice RING finger proteins in growth and stress response still remain unknown. In this study, we identified a novel RING-H2 type of protein, *OsRF1*, which acts as a positive regulator of drought and salt stress responses in rice. The stress tolerance of the *OsRF1*-OE transgenic rice strongly correlated with ABA accumulation and ABA hypersensitivity. We further demonstrated that OsRF1 directly interacts with the clade A protein phosphatase OsPP2C09, a core negative regulatory component of ABA signaling components, and targets for ubiquitination and protein degradation, suggesting that *OsPP2C09* is one of the molecular linkages of *OsRF1* function in ABA-dependent stress tolerance.

Several studies on RING finger proteins in plants have reported that the overexpression of RING-H2 proteins confers drought tolerance to plants by increasing ABA levels in different ways. First, the overexpression of RING-H2 proteins induces the expression of genes related to ABA biosynthesis, increasing endogenous ABA contents. Ko et al. (2006) and Zeng et al. (2013) reported the overexpression of *AtXerico*-induced expression of *AtNCED3* in *Arabidopsis* and *OsNCED* and *OsABA3* in rice under stress conditions (Ko et al., 2006; Zeng et al., 2013). In rice, the overexpression of *OsRHP1* increased *ZEP, NCED*, and *ABA3* expression when challenged with salt and drought stress (Zeng et al., 2014). Second, the overexpression of RING-H2 proteins blocks ABA degradation pathways by diminishing the protein stability of ABA hydrolase and keeping the ABA level at high concentration. In the presence of full-length *ZmXerico*, the protein level of ABA 8-hydroxylase, a key component of ABA catabolism, was significantly decreased in maize protoplast (Brugiere et al., 2017). We found that the overexpression of *OsRF1* enhanced ABA levels by inducing ABA biosynthetic pathway, similar to the cases of *AtXerico* and *OsRHP1* (Figure 2). Under drought stress, the expression of *ZEP*, *ABA4*, and *NCED3* in *OsRF1*-OE was significantly increased than in WT within 1–4 h (Figure 2C). The advanced expression of ABA biosynthetic genes in response to drought stress could result in higher accumulation of ABA in early stress stage, which lets rice cope with drought stress immediately. Consistent with this, *OsRF1*-OE had higher ABA contents than WT and exhibited strong resistance against drought stress (Figures 2A, 4A). The reduced water loss rate (Figures 4C,D) is evidence of the elevated drought tolerance of *OsRF1*-OE *via* ABA accumulation (Figures 2A, 4C,D), since water saving/conservation through stomatal closure is one of the defense mechanisms regulated by ABA in response to drought stress (Osakabe et al., 2014).

The sequence-based analysis of *OsRF1*indicated that OsRF1 contains conserved a RING-H2 domain, suggesting the potential E3 ligase activity of OsRF1 (Figure 1). In agreement with this, the self-ubiquitination assay showed that the OsRF1 protein exhibits E3 ligase activity (Figure 6A, left panel). Our results from the Y2H, BiFC, and Co-IP analyses indicated direct protein-protein interactions between OsRF1 and OsPP2C09 (Figures 5A,B, 6C). Furthermore, the *in vitro* ubiquitination assay revealed that the OsPP2C09 protein was polyubiquitinated in the presence of OsRF1 protein (Figure 6A, right panel). The degradation of PP2CAs *via* the 26S proteasome could be a manner of regulation of ABA signaling, since PP2CA proteins are key negative regulators of ABA signaling (Ma et al., 2009; Park et al., 2009; Santiago et al., 2009). Indeed, the OsPP2C09 protein is less stable in the presence of OsRF1 (Figure 6B). To date, only a few E3 ligases targeting the PP2CA protein for degradation have been reported. With regard to this aspect, Wu et al. (2016) reported that the *Arabidopsis* E3 ligases *RGLG1* and *RGLG5* act as modulators of ABA signaling by mediating PP2C protein degradation (Wu et al., 2016). COP1-mediated ubiquitination of clade A PP2Cs, ABI/HAB group, and *AHG3* triggers protein degradation of these PP2Cs and promote ABA-mediated stomatal closure in *Arabidopsis* (Chen et al., 2021). *OsRF1* could participate in the regulation of ABA signaling and stress responses by controlling the OsPP2C09 protein dynamics under stress conditions. Recently, it was reported that overexpression of OsPP2C09 displayed ABA-insensitive and stress-hypersensitive phenotypes, and that the knock-out mutant of OsPP2C09 showed enhanced stress tolerance and ABA hypersensitivity (Miao et al., 2020; Min et al., 2021). Although the OsPP2C09 protein level is known to be regulated by the 26S proteasome pathway (Miao et al., 2020), the mechanism of PP2CA09 degradation is still unknown. With regard to these reports, we proposed that *OsPP2C09* is a target of *OsRF1*, and that the regulation of OsPP2C09 protein stability could a mode of action of *OsRF1* for conferring drought and salt tolerance.

Besides enhanced drought and salinity tolerance, the overexpression of *OsRF1* in rice displayed growth retardation (Supplementary Figure 3B) and seed-dormant phenotypes (Supplementary Figures 3D,E). Plants develop an adaptation mechanism to restrain growth under unfavorable environmental conditions. Growth-repressing DELLA proteins, key negative regulators of GA signaling, play essential role to permit flexible plant growth against adverse conditions (Achard et al., 2006). It is assumed that the growth phenotype of *OsRF1*-OE rice is closely correlated with DELLA proteins because of higher accumulation of ABA and/or ABA hypersensitivity (Figures 2, 3, 7). To date, whether ABA inhibits GA signaling by stabilizing DELLA proteins to restrain growth under stress conditions is controversial. While Achard et al. (2006) reported that ABA protects GFP-RGA fusion protein from GA-induced degradation (Achard et al., 2006), ABA pretreatment could not affect endogenous RGA levels in the presence or absence of GA (Zentella et al., 2007), and ABA had no effect on the GA-induced degradation of SLN1, a barley DELLA protein (Gubler et al., 2002). In our present study, we examined the endogenous levels of SLR1, a rice DELLA protein, in WT and *OsRF1*-OE rice and found that the SLR1 protein accumulated at a higher level with the ABA treatment (Figures 7A,B). We further demonstrated that the GA-dependent rapid degradation of the SLR1 protein was significantly delayed in the *OsRF1*-OE seedlings compared to WT (Figure 7B). In line with this, the *OsRF1*-OE rice diminished GA response in stimulating leaf sheath elongation (Figure 3). Based on these results, we proposed that *OsRF1* plays an important role in the regulatory circuit between ABA and GA pathway. *OsRF1* not only intensified the ABA signaling pathway by accelerating ABA-induced OsPP2C09 protein degradation, but it also diminished the GA signaling pathway by protecting of the SLR1 protein from GA-induced degradation (Figure 8). As a result, *OsRF1* concurrently functions to restrict growth and enhance stress response (Figure 8). With regard this aspect, it is noteworthy that the GA-induced SLR1 protein degradation was promoted in the *OsPP2C09*-OE transgenic rice compared to the WT plants (Figure 7C). Protein kinases and phosphatases have been suggested to play important roles in the stability of DELLA proteins and GA signaling. A type-1 protein phosphatase, TOPP4, destabilizes *Arabidopsis* DELLA proteins by dephosphorylation (Qin et al., 2014). In rice, a casein kinase, early flowering 1 (EL1) stabilizes the SLR1 protein by phosphorylation (Dai and Xue, 2010). These reports and our data support a potential role of OsPP2C09 in controlling SLR1 protein stability and GA signaling in rice. Miao et al. (2020) recently suggested *OsPP2C09* as an essential player in balancing plant growth and drought tolerance in rice (Miao et al., 2020). The interplay between *OsRF1* and *OsPP2C09*-mediated ABA-dependent pathway for regulating GA signaling and growth response under stress conditions needs to be further elucidated.

In conclusion, we identified *OsRF1*, a new RING E3 ligase in rice that is involved in ABA signaling regulation in response to drought and salt stress. The overexpression of *OsRF1* caused the accumulation of endogenous ABA by enhancing the ABA biosynthesis pathway, conferring drought and salt tolerance to rice. We also revealed the E3 ligase activity of OsRF1 on OsPP2C09 and suggested *OsRF1* as a new member of a complex ABA regulatory network in response to drought and salt stress (Figure 8). Our present results support that OsRF1 positively regulates ABA signaling by targeting OsPP2C09 for degradation, which suggests that OsRF1 may act genetically upstream of OsPP2C09. To our knowledge, this is the first report about a RING-H2 E3 ligase in rice that explains how it contributes to ABA signaling and growth regulation under stress conditions. Although further questions on how *OsRF1* genetically interacts with *OsPP2C09*, *SLR1*, or other ABA signaling components still remains, this finding could give a new insight into a new strategy to conquest drought and salinity problems by providing an extensive understanding of drought and salt stress response in plant.

## Data Availability Statement

The original contributions presented in the study are included in the article/Supplementary Materials, further inquiries can be directed to the corresponding author/s.

## Author Contributions

ISY and SK conceptualized and wrote the original draft. ISY and S-IP revised the manuscript. SK, S-IP, HK, MHC, and JSS performed the experiments and data analysis. MHN and JHC analyzed ABA content. B-GK and K-HK reviewed the manuscript. ISY supervised this study. All authors contributed to manuscript revision, read, and approved the manuscript.

## Conflict of Interest

The authors declare that the research was conducted in the absence of any commercial or financial relationships that could be construed as a potential conflict of interest.

## Publisher’s Note

All claims expressed in this article are solely those of the authors and do not necessarily represent those of their affiliated organizations, or those of the publisher, the editors and the reviewers. Any product that may be evaluated in this article, or claim that may be made by its manufacturer, is not guaranteed or endorsed by the publisher.

## Footnotes

1
https://www.expasy.org/resources/compute-pi-mw

2
http://www.cbs.dtu.dk/services/TMHMM-2.0/

3
https://prosper.erc.monash.edu.au/
