# Supplementary material for: The Rice Abscisic Acid-Responsive RING Finger E3 Ligase OsRF1 Targets OsPP2C09 for Degradation and Confers Drought and Salinity Tolerance in Rice
Source: Front Plant Sci. 2022 Jan 13;12:797940. doi: 10.3389/fpls.2021.797940 (PMC8792764; doi:10.3389/fpls.2021.797940)
Supplement: Supplementary file 1 [file Data_Sheet_1.pdf]

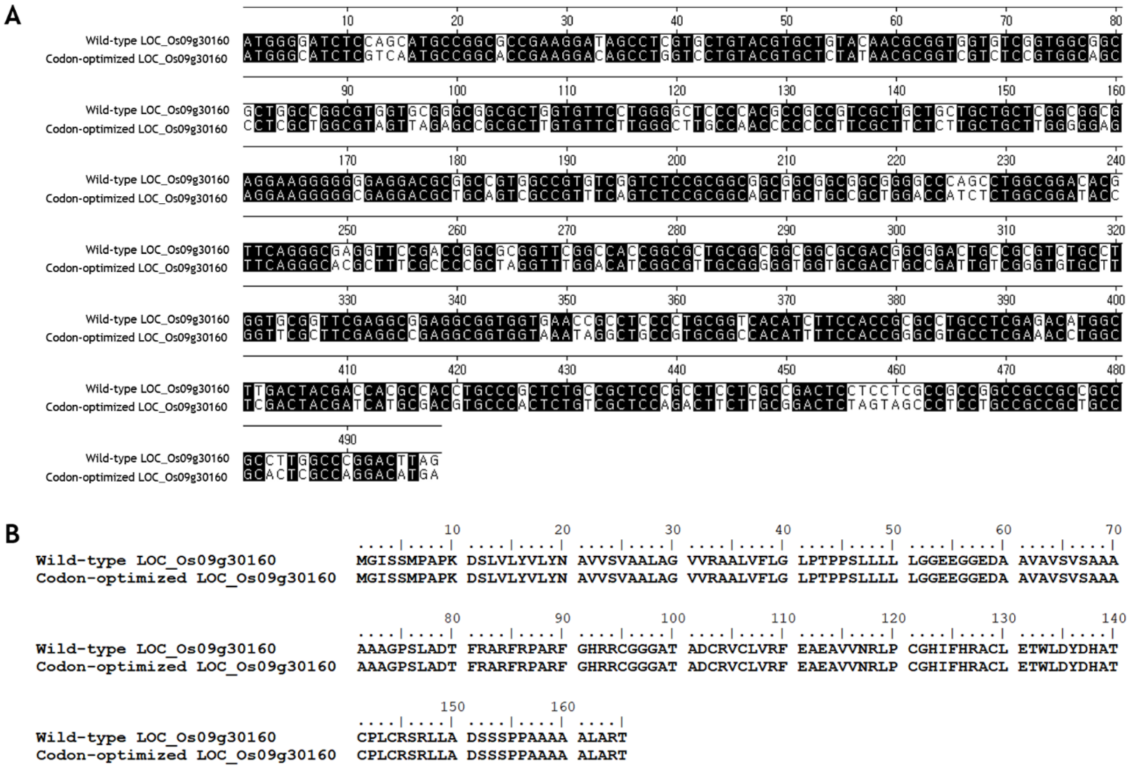

Supplementary Figure S1 Nucleotide and amino acid sequence alignments of wild type and codon-optimized synthetic ORF of *OsRF1* (LOC_Os09g30160) gene. **(A)** Nucleotide sequence alignments. **(B)** Amino acid sequence alignments.

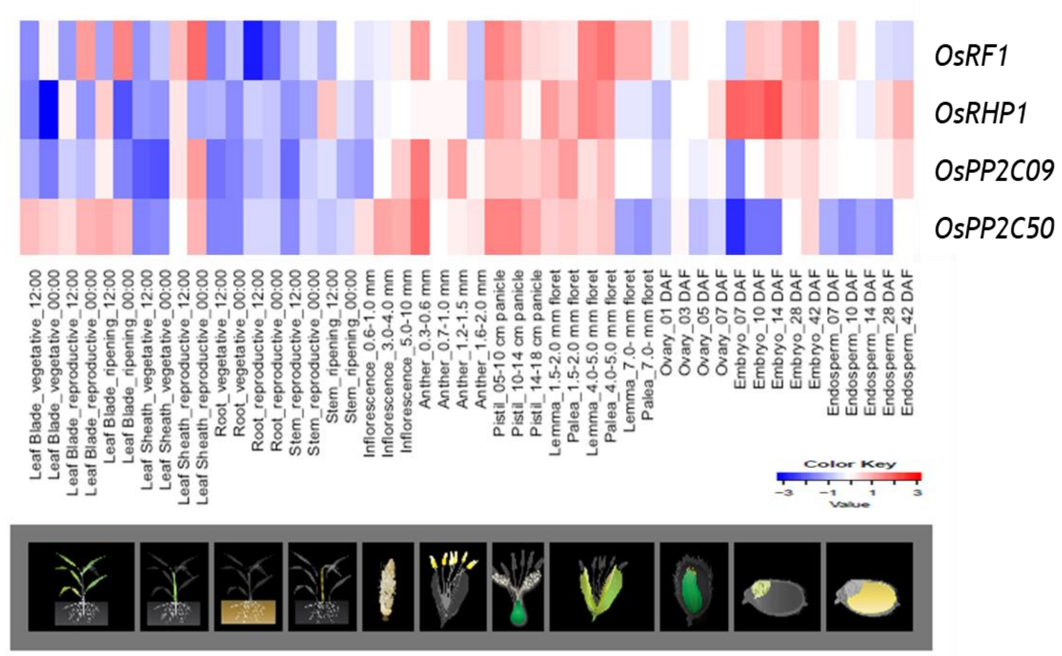

Supplementary Figure S2 Spatio-temporal gene expression analysis of *OsRF1*, *OsRHP1*, *OsPP2C09*, and *OsPP2C50* in various tissues/organs at different developmental stages. Heatmap of normalized Cy3 signal intensity values for indicated genes was constructed according to the RiceXPro database.

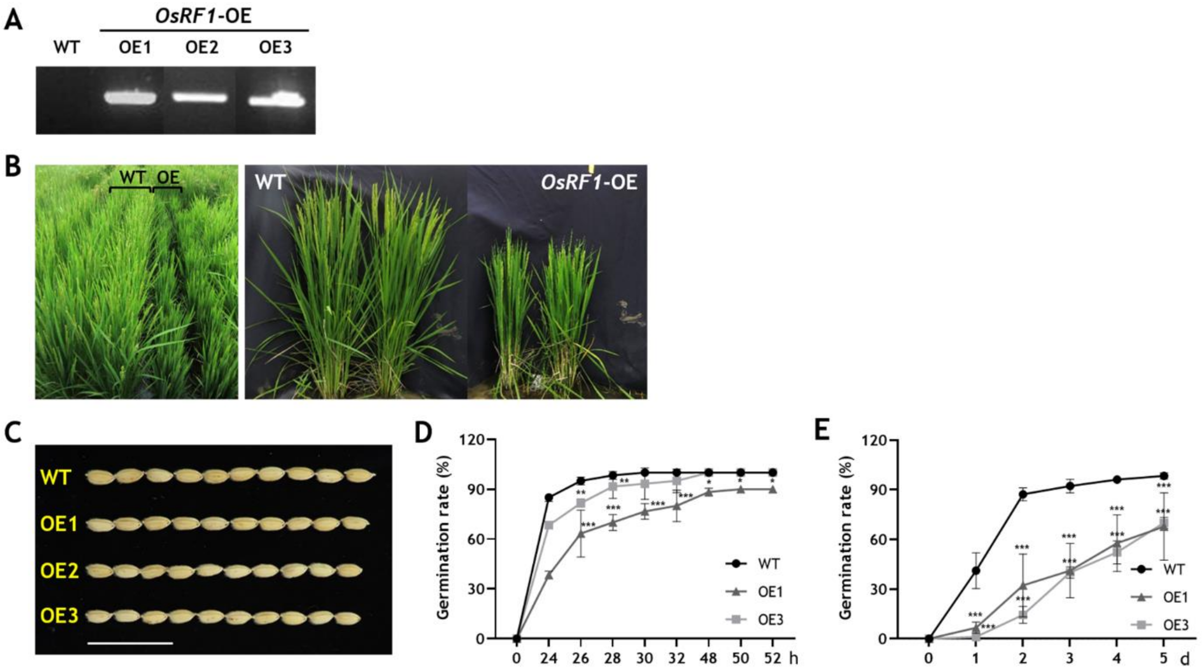

Supplementary Figure S3 Characterization of growth phenotype and seed dormancy of *OsRF1*-overexpressing transgenic and WT rice plants. **(A)** RT-PCR analysis of *OsRF1*-overexpressing (*OsRF1*-OE) transgenic rice lines using a primer set (OsRF1M-F and OsRF1M-R) in **Supplementary Table S1**. **(B)** Growth phenotypes of the *OsRF1*-OE plants at heading stage under paddy field conditions. **(C)** Photographs of OE and WT seeds with husk (n=10). Bar = 2 cm. Germination rates of mature seeds **(D)** and 41 DAH seeds **(E)** at 30 °C in the dark. Data represent mean (± SD) from three independent experiments with two biological replicates (n=30). Two-way ANOVA and Fisher's LSD test were performed comparing with WT seeds as controls. *, $P < 0.05$; **, $P < 0.01$; ***, $P < 0.001$.

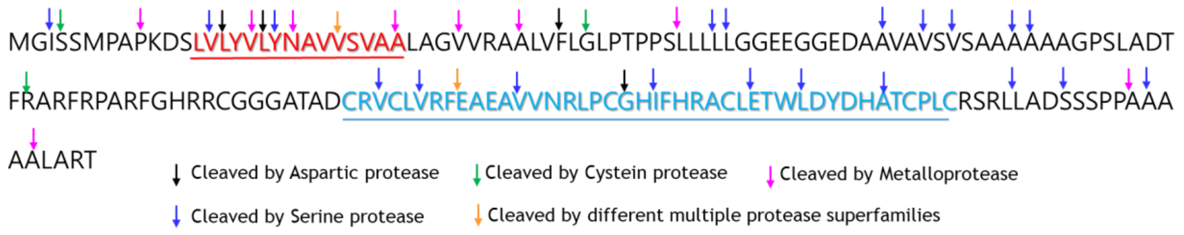

Supplementary Figure S4 Prediction of cleavage sites of different protease families using PROSPER server. Red and blue underlines denote transmembrane domain and RING-H2 domain, respectively.

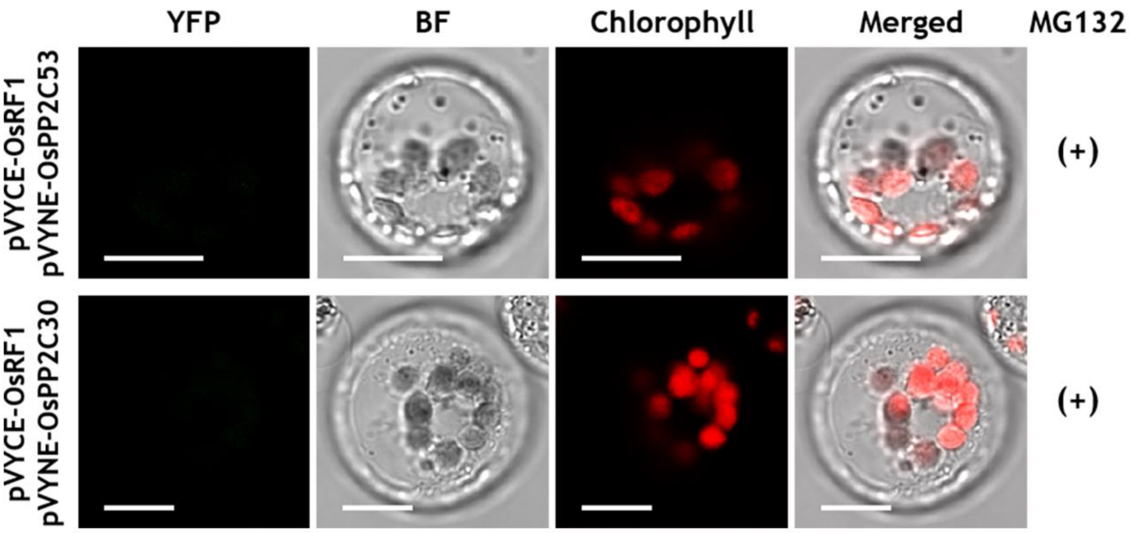

Supplementary Figure S5 Protein-protein interaction assays by BiFC in rice protoplasts. Construct pairs of pVYCE-OsRF1 and pVYNE-OsPP2C53 or pVYNE-OsPP2C30 were transiently co-expressed in rice protoplasts with MG132 and these were observed by confocal microscopy. The YFP fluorescence (green), chlorophyll autofluorescence (red), bright field, and the combined images were visualized under a confocal microscope. Bar = 10 μm.

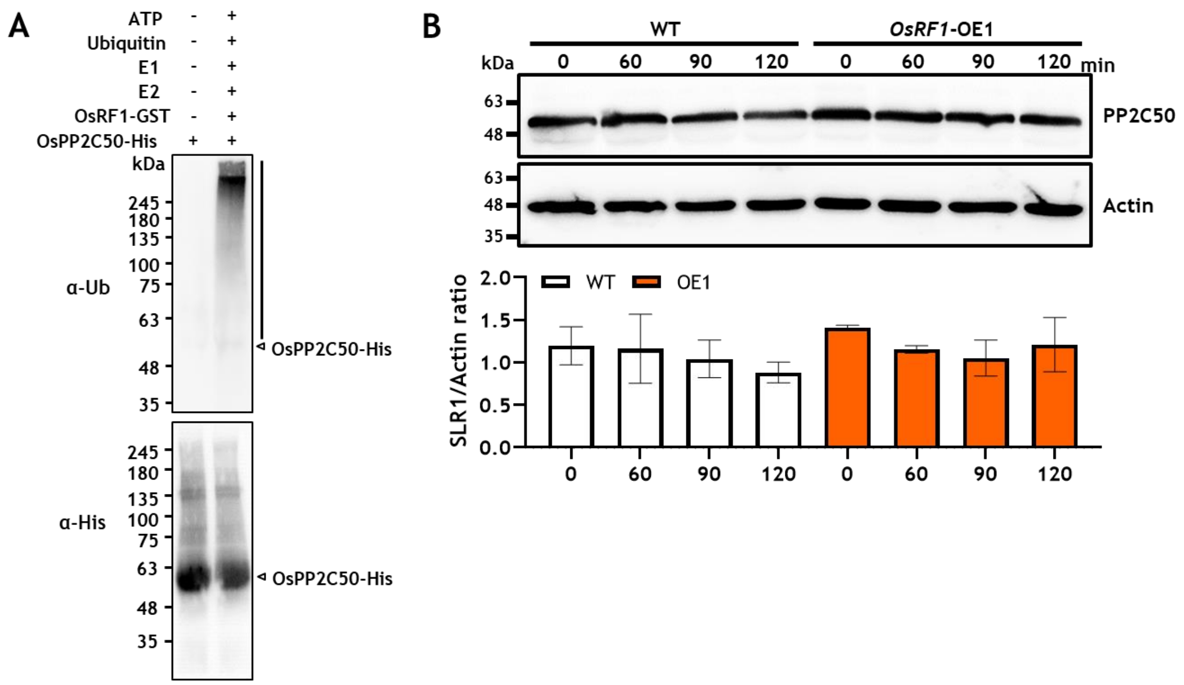

Supplementary Figure S6 *In vitro* ubiquitination and cell free degradation assay of OsPP2C50. (A) *In vitro* ubiquitination of OsPP2C50 by OsRF1. The purified OsRF1-GST was incubated with OsPP2C50-His protein in the presence or absence of human E1, human E2 and bovine ubiquitin (Ub), and incubated at 30 °C for 2 h. The reaction samples were mixed with 5X SDS-PAGE loading buffer, resolved in SDS-PAGE, and immunoblotted with anti-ubiquitin, anti-GST, and anti-His antibodies. The polyubiquitinated proteins were indicated as black bar. (B) Cell-free degradation assay of OsPP2C50. Protein extracts were prepared from seedlings of wild type and OsRF1-OE lines and incubated with purified OsPP2C50-His recombinant protein at 28 °C supplemented at indicated time points. Anti-His antibody was used to detect OsPP2C50-His protein. Quantification of anti-OsPP2C50-His and anti-Actin signal intensity using Image J software. The OsPP2C50 protein levels were normalized by anti-Actin intensity. Representative data from two independent experiments.

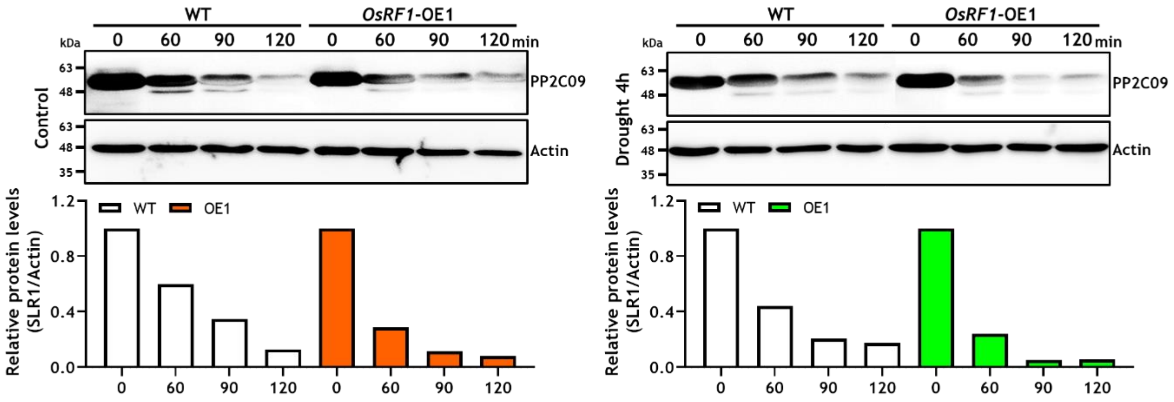

Supplementary Figure S7 Cell-free degradation assay of OsPP2C09 in drought stressed plants. Protein extracts were prepared from seedlings of wild type and *OsRF1*-OE lines exposed to drought stress for 4 h and incubated with purified OsPP2C09-His at 28 °C supplemented at indicated time points. Anti-His antibody was used to detect OsPP2CA-His protein. Quantification of anti-OsPP2CA-His and anti-Actin signal intensity using Image J software. The protein levels of His-PP2C09 in WT or *OsRF1*-OE1 extracts with the indicated treatments at 0 min were set as 1.

Supplementary Table S1. Primer sequences used in this study.

Oligo name	Oligonucleotide sequences (5′-3′)
UBQ5-F	AGAAGCGCAAGAAGAAGACG
UBQ5-R	GCGTCGTCCACCTTGTAGA
OsRF1-F	TGATCGCCATTGTTCAGCAAG
OsRF1-R	GGTGCATGACCAAGTACAGA
OsRF1M-F	GGACAGCCTGGTCCTGTACGTG
OsRF1M-R	CACGTCGCATGATCGTAGTCG
OsZEP-F	TATGACCGACCACCTACTTT
OsZEP-R	GGTAACCATCCTCAATAGCC
OsABA4-F	CCTATCCTGGACTCCCGACA
OsABA4-R	GGCACAGAGAAACCGAATCGC
OsNCED3-F	GGAACGCGTGGGAGGACGACG
OsNCED3-R	GGCGGATCTCGGAGAGCACGC
OsABA3-F	TCTTCATAGAGTCTCCCACA
OsABA3-R	GTAACTTTGCACCTCATACC
